# First experience of wave speed guided point-by-point cavo-tricuspid isthmus ablation for typical atrial flutter

**DOI:** 10.1007/s10840-023-01531-x

**Published:** 2023-03-21

**Authors:** Ermengol Vallès, Jesús Jiménez, Carlos González, Fátima Zaraket, Oriol Rodríguez, Laia Llorca, Ignasi Anguera, Andrea di Marco, Roger Fan, Benjamin Casteigt

**Affiliations:** 1 Electrophysiology Unit, Cardiology Department, Hospital del Mar, Institut Hospital del Mar Investigacions Mèdiques (IMIM), Universitat Pompeu Fabra, 25-27 Passeig marítim de la Barceloneta, 08003 Barcelona, Spain; 2 Electrophysiology Unit, Cardiology Department, Hospital de Bellvitge, Universitat Central de Barcelona, Barcelona, Spain; 3 Stony Brook University School of Medicine, Stony Brook, NY USA

Ablation procedures for cavo-tricuspid isthmus (CTI)–dependent typical atrial flutter (AFL) have classically been guided by fluoroscopy, but nowadays, increasing evidence suggests that electro-anatomic mapping systems (EAM) may be useful to diminish fluoroscopy and guide ablation, targeting high voltage areas [1–3]. Areas of slow conduction in the CTI are the primary substrate of the macroreentrant circuit [4, 5]. We have tested a strategy of CTI ablation targeting areas of slow conduction and high voltage, identified with the omnipolar vectors (OV: maximum voltage vector determined from a triangular set of three electrodes, automatically selected from all directions), which allow obtaining improved voltage maps and detecting wave speed.

We conducted a conceptual pilot study in patients undergoing CTI RF ablation for AFL. We prospectively evaluated a novel strategy where ablation was guided by OV delineating sites of slow wave speed and high voltage. All procedures were performed with Ensite EAM, HD-Grid multipolar diagnostic catheter, and Tacti-cath contact-force irrigated tip ablation catheter (Abbott). We identified the areas with the slowest wave speed (wave speed values < 30% of average value in the CTI); afterwards, we identified the areas with the highest voltage (voltage values > 70% of average voltage in the CTI). Finally, areas with overlap between slow wave speed and high voltage were identified (Fig. 1). Acute success was defined by the achievement of bidirectional block in the CTI. RF ablation was first attempted at the focal sites of confluence between slow wave speed and high voltage. If bidirectional conduction block was not achieved, the second step consisted of ablation of the remaining sites of slow wave speed. If unsuccessful, the third step was ablation of the remaining sites of high voltage. Finally, if bidirectional CTI block was not achieved with the aforementioned steps, a CTI line was performed until success.

**Fig. 1:**
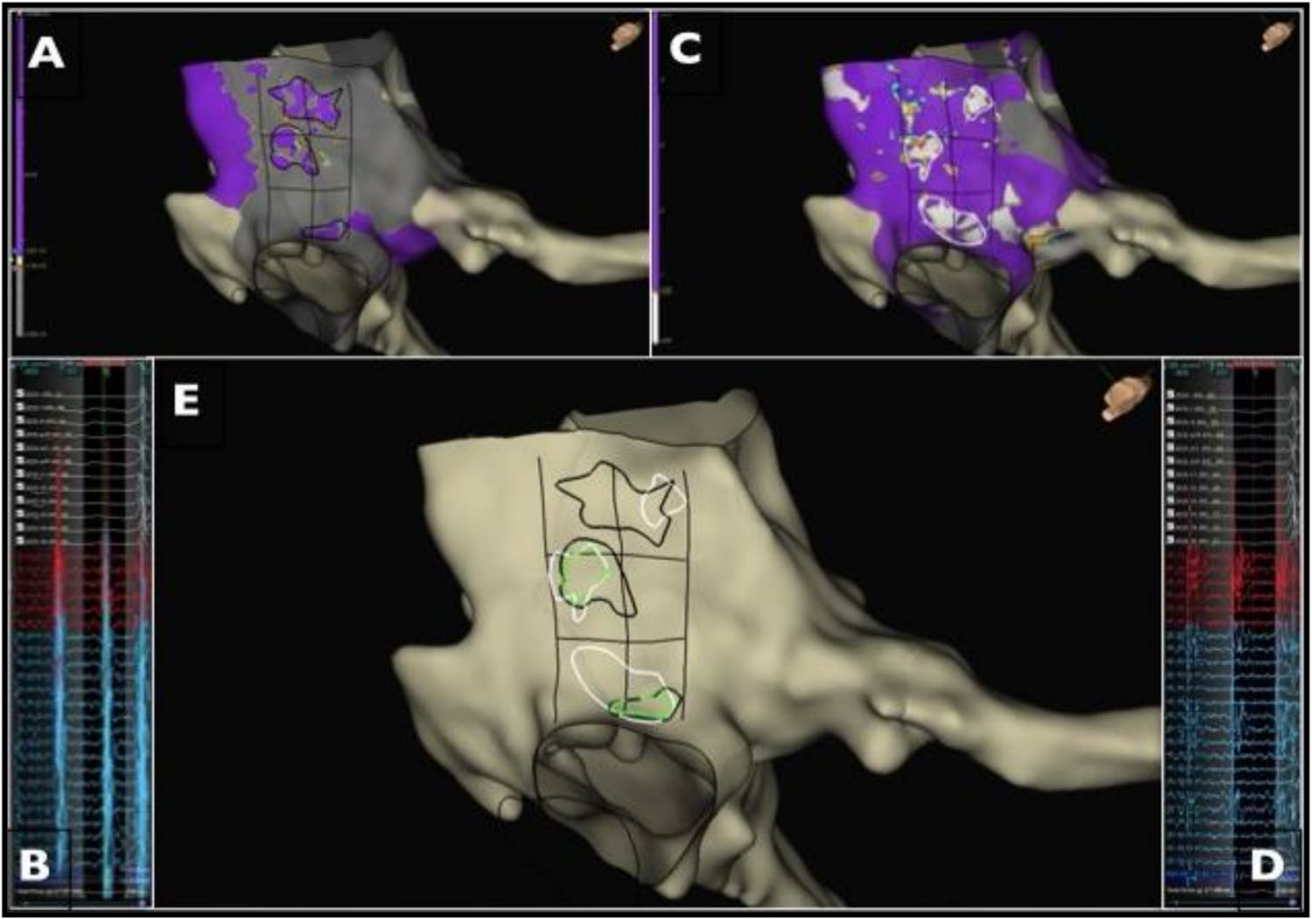
LAO caudal view, showing the CTI. Panel **A** shows the areas of high voltage encircled in black. Panel **B** displays the prominent EGMs in those high voltage areas. Panel **C** shows the areas of slow wave speed encircled in white. Panel **D** displays the fractionated EGMs in those low wave speed areas. Paned **E** shows all the previous areas and highlights confluent sites encircled in green

A total of 26 patients were included (mean age 68 ± 12 years, 88% male). The procedure was performed during stimulation at 600 ms cycle length from the proximal coronary sinus in the 61% of patients and during AFL in the rest of the 39%. Mean procedure time was 83 ± 19 min. Median fluoroscopy time was 0 min (interquartile range 0–1), and median radiation dose was 0 mGray (interquartile range 0–13). Zero fluoroscopy was used in 62% of the procedures. Targeted areas of slow wave speed in the CTI had a velocity of 0.57 ± 0.25 mm/ms, compared to the average velocity of the whole RA (0.91 ± 0.27 mm/ms). Targeted high voltage areas in the CTI were also consistently higher (3 ± 0.95 mV) than the average voltage in the RA (2.2 ± 0.6 mV). Concerning ablation, a median of 13 RF lesions (interquartile range 9–16) was performed and mean RF time was 349 ± 149 s. Quality of lesions was assessed by LSI, which averaged 5 ± 0.5. Regarding procedure success, CTI block was obtained with ablation at the slow-wave speed/high voltage confluence areas in 16 patients (53.9%), 10 patients (38.5%) patients needed additional applications in areas of isolated slow wave speed, 1 patient needed ablation in areas of isolated high voltage, and only 1 patient (3.8%) needed the performance of a complete CTI line. Thus, in 24/26 patient (92%), ablation at areas of slow wave speed achieved complete CTI block. During a mean follow-up of 12 ± 6 months, 24-h Holter and symptomatic event recorder were performed in 11 and 1 patients, respectively, because of isolated palpitations, with 1 clinical recurrence (3.8%).

The availability of new EAM catheters and systems capable of identifying regions of local slow wave speed opens a new horizon. Traditionally, stable reentrant arrhythmias have been treated by targeting the weakest point, the isthmus of the circuit [6]. Our results indicate that these slow CTI conduction pathways should be considered as unique conduction corridors in the CTI and cornerstones for typical AFL maintenance. These areas are surrounded by large muscle fibers, which are often detected on omnipolar mapping as high voltage areas.

In conclusion, ablation in areas of slow conduction achieves CTI bidirectional block in more than 92% of patients and should be targeted preferentially. This approach may be able to diminish procedure, fluoroscopy, and RF burden. Although, these observations are made in basis of a non-controlled study and warrant confirmation in an ongoing, larger, prospective randomized study (clinicatrials.gov NCT05709795).

## Data Availability

The data that support the findings of this study are available from the corresponding author upon reasonable request.

## Declarations

This study was approved by our institution’s ethical committee on human research.
